# Non-clinical symptoms associated with polycystic ovary syndrome: evidence from a comparative study in Bangladesh

**DOI:** 10.7189/joghtemplate

**Published:** 2026-09-25

**Authors:** KM Tanvir, Syed Shahadat Hossain, Humayara Mahjebin Turin, Mishkat Mahiuddin, Tasnim Ara, Mahanaz Faiza, Merajul Islam, Arafat Sabbir, Md Rasel Biswas, Tarikul Islam, Tahseen Khan, Saraban Tahura Ether, Ismat Ara Hossain, Anisuddin Ahmed

**Affiliations:** 1 Institute of Statistical Research and Training, University of Dhaka, Dhaka, Bangladesh; 2 Civil and Environmental Engineering, Islamic University of Technology, Gazipur, Bangladesh; 3 Shaheed Suhrawardy Medical College and Hospital, Dhaka, Bangladesh; 4 International Centre for Diarrhoeal Disease Research, Dhaka, Bangladesh; 5 Army Medical College, Joshore, Bangladesh

**Keywords:** polycystic ovary syndrome, PCOS, symptoms, reproductive health, Bangladesh

## Abstract

**Background:**

Polycystic ovary syndrome (PCOS) is a common endocrine disorder that occurs in women of reproductive age and is linked to pregnancy, metabolic, and psychological complications. Although it is very prevalent, a significant percentage of the cases are not diagnosed, because of poor awareness and access to diagnostic services. Identifying non-clinical symptom patterns associated with PCOS may support the development of symptom-based risk assessment approaches and facilitate earlier clinical evaluation of women at increased risk of the condition. The objective of the study was to examine differences in non-clinical, self-reported, and observable symptom profiles between women with and without PCOS in Bangladesh.

**Methods:**

A cross-sectional study was carried out on 546 women between the ages of 15 and 30 years in Bangladesh, 273 with physician-diagnosed PCOS and 273 controls. χ^2^ tests were conducted on bivariate analyses and multivariate logistic regression was conducted to determine the possible symptoms of PCOS with age and body mass index as confounding variables.

**Results:**

There were a number of symptoms that were significantly different in the two groups. The strongest associations with PCOS in multivariate analysis were irregular menstrual cycles (odds ratio (OR) = 4.93), unexpected weight gain (OR = 4.32), and hair growth in the jawline (OR = 4.25). The condition was also significantly related to self-reported sleep difficulty (OR = 2.62) and waist circumference greater than hips (OR = 2.47). Conversely, the symptoms that were frequently reported, including acne, oily skin, loss of hair, anxiety or stress, hair growth in upper lip, thigh, and upper arm, and family history of PCOS were not statistically significant after adjustments.

**Conclusions:**

Several observable and self-reported symptoms were associated with PCOS among Bangladeshi women. These findings provide a basis for future research aimed at developing and validating symptom-based risk assessment approaches for PCOS.

Polycystic ovary syndrome (PCOS) is considered to be one of the most widespread endocrine disorders in women of reproductive age, and its prevalence is estimated at around 9.2% worldwide [1]. The disorder is linked to various reproductive, metabolic, and psychological complications, which may have a significant impact on the health and quality of life of women [2]. Although PCOS is very common and clinically important, early diagnosis of the condition is a significant challenge and according to the World Health Organization, nearly 70% of the affected women are yet to be diagnosed in the world [3].

PCOS is a chronic disorder with diverse symptoms and severe health complications. Commonly reported symptoms include irregular or absent menstrual cycles, excessive hair growth (hirsutism), acne, thinning of scalp hair, and unexpected weight gain [4–6]. PCOS also has significant health consequences, as it is one of the most common causes of infertility and is associated to adverse pregnancy outcomes, including gestational diabetes, pregnancy-induced hypertension, preeclampsia, and a higher risk of preterm birth and low birth weight [7–9]. Moreover, PCOS is strongly related to metabolic disruption including insulin resistance, obesity, and dyslipidaemia that predispose to type 2 diabetes and cardiovascular disease in the long-term [10–12]. In addition, women with PCOS tend to have psychological issues, such as anxiety, depression, and low self-esteem, which are influenced by the observable symptoms, fertility issues, and the need to manage chronic disorder [13–15].

Though PCOS is incurable, its symptoms and risks can be well controlled by ensuring that it is identified in time and managed properly [16,17]. Early diagnosis enables the adoption of lifestyle modifications, specific medical care, and frequent follow-ups, which may greatly enhance the reproductive outcomes and minimise the risk of the long-term complications [18,19]. Nevertheless, the early diagnosis is still quite a challenge, as the PCOS diagnosis is complex in nature and usually involves a mix of clinical examination, biochemical analysis, and imaging [20,21]. The most popular diagnostic methods, including the Rotterdam criteria, are based on the detection of ovulatory dysfunction, hyperandrogenism, and polycystic ovarian morphology with the help of ultrasound [22].

The diagnostic processes tend to rely on the availability of special healthcare services, trained practitioners, and laboratory facilities, which are not always easily accessible in most low- and middle-income countries [23]. Moreover, social and cultural barriers, as well as the lack of awareness about the initial symptoms, also contribute to the further delay of care-seeking behaviour in women [24,25]. As a result, a significant percentage of women are not diagnosed in a timely manner, which results in late treatment and the risk of developing adverse reproductive and metabolic outcomes. Due to the difficulties related to clinical diagnosis, the role of symptoms as a possible foundation of early PCOS identification is becoming increasingly popular [26]. Observable and self-reported characteristics are easy to evaluate, need few resources, and may be integrated into community-based screening or digital health applications. These could help to identify PCOS earlier, promote prompt healthcare-seeking behaviour, and decrease the number of undiagnosed cases [27].

Despite the large amount of research that has been conducted on the clinical and biochemical features of PCOS, the majority of studies have been conducted to validate the diagnosis using hormonal tests, imaging, or clinical criteria. Although these methods are necessary to make a conclusive diagnosis, they can be costly in terms of resources and are not available to all women. Simultaneously, despite the fact that a broad spectrum of symptoms related to PCOS have been reported, there is still a lack of evidence on the relative significance of these symptoms when they are taken into account together. In addition, PCOS is highly heterogeneous in the manifestation of symptoms, and the differences in ethnicity, lifestyle, and environmental factors are observed in different populations. Indicatively, East Asian women with PCOS are less likely to have hirsutism and more likely to experience menstrual problems, whereas South Asian, Hispanic, Middle Eastern, and Black women are more likely to have higher androgen levels, increased insulin resistance, and obesity [28]. Nevertheless, no proper evidence is available focusing on Bangladeshi women, although the issue of PCOS is increasingly becoming a burden in this nation.

To address these gaps, the current study will compare the symptom profiles of women with and without PCOS to determine which observable and self-reported symptoms are most strongly associated with the condition among Bangladeshi women. By fulfilling these goals, the study may help inform the development of symptom-based risk assessment approaches for identifying women at increased risk of PCOS, particularly in resource-limited settings.

## METHODS

### Study setting and population

This study was cross-sectional in design, and findings are reported in accordance with the STROBE reporting guidelines for observational studies (Table S1 in the **Online Supplementary Document**). This study was carried out to determine the difference in symptom profile between women with and without PCOS. Data were collected from six tertiary healthcare institutions located in different regions of Bangladesh, namely Dhaka Medical College Hospital, Mohammadpur Fertility Center, Shaheed Suhrawardy Medical College and Hospital, Army Medical College Jashore, Institute of Child and Mother Health (Matuail), and Rajshahi Medical College Hospital. The data collection was conducted from 28 May to 28 August 2025.

The women who were aged between 15 and 30 years and had undergone a formal clinical diagnosis of PCOS were eligible to be included. The cases were women who had received a physician-confirmed diagnosis of PCOS prior to recruitment. Controls were women who had previously undergone clinical evaluation and had been determined by an attending physician not to have PCOS. Therefore, both case and control status, including adolescent participants, were based on prior physician assessment by modified Rotterdam criteria rather than a diagnostic evaluation conducted specifically for this study. Pregnant women and those with any significant chronic illness were not allowed to participate. Recruitment was done in a sequential manner, with eligible women being enrolled one after another as they reported at the study sites until the target sample size was reached.

### Data collection

The structured questionnaire was prepared on an iterative development process. The initial version of the instrument was prepared by a comprehensive review of relevant literature and discussions with clinicians. It was later updated under the advice of a gynaecologist who has expertise in PCOS so that it is clinically appropriate. The data was collected using interviewer-administered questionnaires with six trained physicians and each session took about 10 to 15 minutes. Kobo Toolbox was used to record responses directly into a digital platform to minimise the possibility of data entry errors. All the completed questionnaires were thoroughly checked in terms of completeness and logical consistency. During the data collection, operational indicators such as the time of starting the interview, the duration of the interview, and the location of the interview were observed. Moreover, a sample of the participants was randomly contacted through telephone in order to confirm the selected answers. Any discrepancies that were found in these processes were resolved by conducting repeat interviews.

### Sample size determination

The required sample size was estimated for a comparative study assessing differences in exposure proportions between women with PCOS and controls. According to the previous evidence, the prevalence of key exposures was assumed to be 25 percent in women with PCOS and 15 percent in controls. The minimum sample size was determined as 248 participants per group using the standard formulae of sample size in comparative studies with 95% confidence level and 80% statistical power [29]. An extra 10% was included to cover the possibility of non-response and incomplete data, and this gave a target sample of about 273 participants per group. Finally, 546 participants (273 PCOS and 273 non-PCOS) were recruited, which is slightly more than the necessary sample size.

### Measures and variable definitions

Information was collected across three broad domains: sociodemographic characteristics, symptom-related variables, and reproductive health indicators. The main outcome measure was PCOS status, which was either diagnosed or non-diagnosed according to the previous medical confirmation.

Symptom-related variables included self-reported experiences of conditions commonly associated with PCOS. Participants were asked to indicate the frequency of symptoms such as hair loss, unexpected weight gain, skin darkening, acne, oily skin, mood swing, anxiety or stress, fatigue, sleep disturbances, and menstrual cramps. Each item was measured using a four-point Likert scale and for analytical purposes, responses indicating ‘frequently’ or ‘regularly’ were classified as the presence of the symptom, whereas responses of ‘never’ or ‘occasionally’ were grouped as absence of the symptom.

Indicators of hirsutism were assessed using visual references depicting hair growth in specific body regions, including the upper lip, jawline, upper arm, and thigh. Participants classified the extent of hair growth into three categories: no hair, a few scattered hairs, or grouped hair growth. For analytical purposes, the latter two categories were combined and treated as the presence of hair growth. Additional variables included menstrual regularity, family history of having PCOS, waist circumference greater than hips, pregnancy history, and body mass index (BMI), which was classified as underweight (<18.5 kg/m^2^), normal weight (18.5–24.9 kg/m^2^), and overweight (≥25.0 kg/m^2^). Sociodemographic variables included age, grouped into three categories (15–20, 21–25, and 26–30 years), education level, marital status, and employment status.

### Statistical analysis

No missing data were identified in the data set, and all 546 recruited participants were included in the final analysis. Multivariate logistic regression analysis was conducted to identify symptoms associated with PCOS. Adjusted odds ratios (ORs) and 95% confidence intervals (CIs) were calculated after controlling the potential confounders. Before multivariate modelling, bivariate analyses were conducted using the χ^2^ test to evaluate the difference in symptoms between women with and without PCOS. Multicollinearity among predictors was assessed using variance inflation factors (VIF). A sensitivity analysis was conducted in which symptom variables originally measured on Likert scales were retained in their original categorical form and included in the logistic regression model to evaluate the impact of dichotomisation. The conventional level of statistical significance was used at *P* < 0.05.

## RESULTS

The two groups were similar in terms of age, education, and reproductive history. Nevertheless, notable variations were found in BMI where overweight status was more prevalent in women with PCOS. The groups also differed in marital status with more married women in the PCOS group. There were also differences in employment patterns, where more students in the non-PCOS group and more employed and homemaker women in the PCOS group (**Table 1**).

**Table 1:** Background characteristics of the respondents where values are presented as frequency and percentage

Variables	Category	PCOS (n = 273)	Non-PCOS (n = 273)	
Age, in years	15–18	19 (6.96)	31 (11.36)	
	19–22	60 (21.98)	62 (22.71)	
	23–26	100 (36.63)	98 (35.90)	
	27–30	94 (34.43)	82 (30.04)	
BMI status	Underweight	10 (3.66)	45 (16.48)	
	Normal	149 (54.58)	166 (60.81)	
	Overweight	114 (41.76)	62 (22.71)	
Education	No or primary	41 (15.02)	57 (20.88)	
	Secondary or higher secondary	90 (32.97)		79(28.94)	
	Graduate or higher	142 (52.01)	137 (50.18)	
Marital status	Unmarried	93 (34.07)	177 (64.84)	
	Married	180 (65.93)	96 (35.16)	
Employment	Student	94 (37.90)	159 (58.24)	
	Employed	73 (29.44)	64 (23.44)	
	Homemaker	81 (32.66)	50 (18.32)	
Have you ever been pregnant?	Yes	90 (32.97)	70 (25.64)	
	No	183 (67.03)	203 (74.36)	

BMI – body mass index, PCOS – polycystic ovary syndrome

Several key symptoms showed significant differences between the two groups (**Table 2**). Women with PCOS were significantly more likely to report irregular menstrual cycles, unexpected weight gain, and darkening of the skin (all *P* < 0.001). A family history of having PCOS was also more common among affected women (*P* = 0.001). Androgen-related dermatological features were markedly higher among women with PCOS, including hair presence in the upper lip, jawline, upper arm, and thigh (all *P* < 0.001), with the largest difference observed for hair presence in the jawline. In addition, women with PCOS more frequently reported anxiety or stress (*P* = 0.002) and sleep difficulty (*P* < 0.001), and a higher proportion had a waist circumference greater than hips (*P* < 0.001). In contrast, symptoms such as frequent acne breakouts, oily skin, fatigue, and menstrual cramps did not show statistically significant differences between the two groups.

**Table 2:** Bivariate comparison of symptomatic characteristics by PCOS status

Variable	Category	PCOS (%)	Non-PCOS (%)	*P*-value
Family History of having PCOS	No	227 (47.39)	252 (52.61)	0.001
	Yes	46 (68.66)	21 (31.34)	
Irregular Period	No	60 (23.81)	192 (76.19)	<0.001
	Yes	213 (72.45)	81 (27.55)	
Darkening Skin	No	130 (40.37)	192 (59.63)	<0.001
	Yes	143 (63.84)	81 (36.16)	
Frequent Unexpected Weight Gain	No	82 (28.47)	206 (71.53)	<0.001
	Yes	191 (74.03)	67 (25.97)	
Frequent Hair Loss	No	21 (37.50)	35 (62.50)	0.048
	Yes	252 (51.43)	238 (48.57)	
Frequent Acne Breakouts	No	104 (45.81)	123 (54.19)	0.099
	Yes	169 (52.98)	150 (47.02)	
Frequent Oily Skin	No	87 (44.85)	107 (55.15)	0.074
	Yes	186 (52.84)	166 (47.16)	
Frequent Anxiety/Stress	No	36 (36.00)	64 (64.00)	0.002
	Yes	237 (53.14)	209 (46.86)	
Frequent Fatigue	No	34 (42.50)	46 (57.50)	0.146
	Yes	239 (51.29)	227 (48.71)	
Frequent Sleep Difficulty	No	85 (35.56)	154 (64.44)	<0.001
	Yes	188 (61.24)	119 (38.76)	
Menstrual Cramps	No	91 (45.73)	108 (54.27)	0.131
	Yes	182 (52.45)	165 (47.55)	
Hair presence in upper Lip	No	113 (37.67)	187 (62.33)	<0.001
	Yes	160 (65.04)	86 (34.96)	
Hair presence in Jawline	No	152 (37.91)	249 (62.09)	<0.001
	Yes	121 (83.45)	24 (16.55)	
Hair presence in Upper Arm	No	205 (45.76)	243 (54.24)	<0.001
	Yes	68 (69.39)	30 (30.61)	
Hair presence in Thigh	No	200 (45.15)	243 (54.85)	<0.001
	Yes	73 (70.87)	30 (29.13)	
Waist circumference greater than hips	No	111 (38.54)	177 (61.46)	<0.001
	Yes	162 (62.79)	96 (37.21)	

BMI – body mass index, PCOS – polycystic ovary syndrome

**Figure 1:**
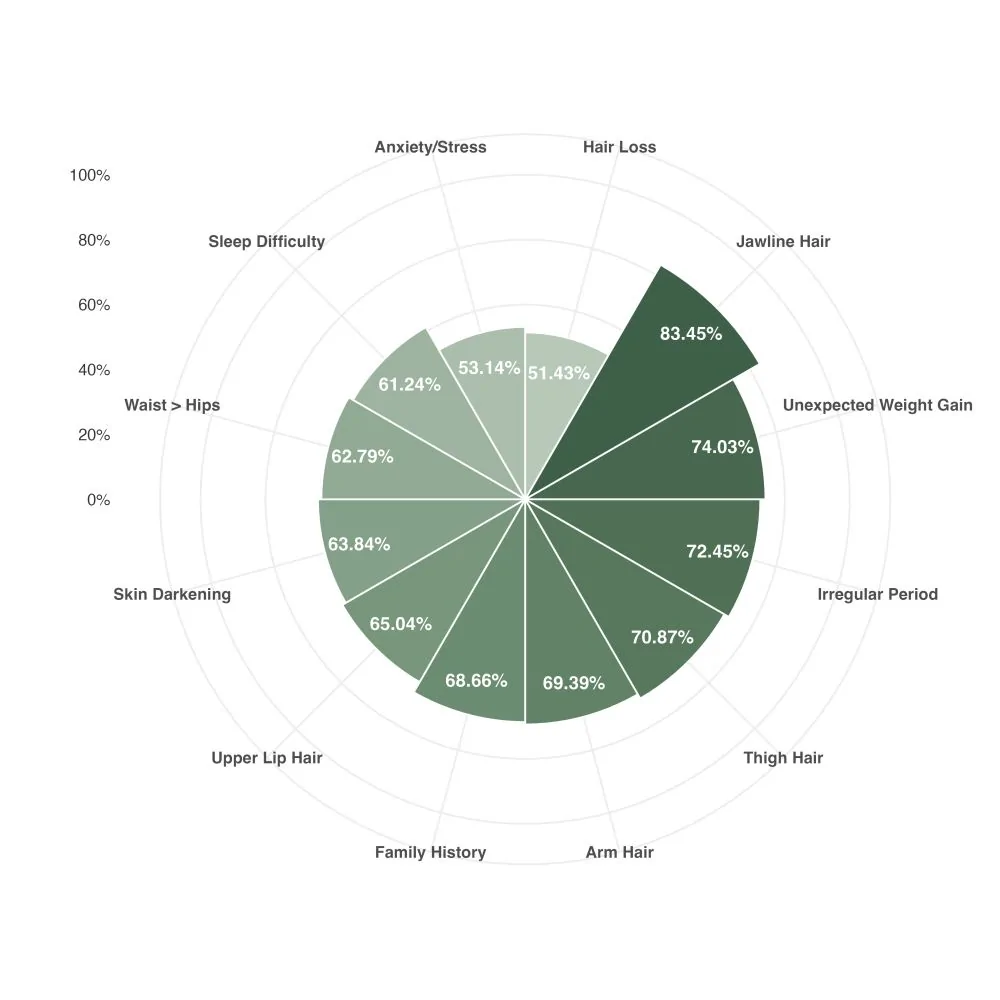
Prevalence of polycystic ovary syndrome (PCOS) by presence and absence of selected symptomatic characteristics where symptoms are ordered in descending order based on the magnitude of difference in PCOS prevalence between groups.

Across all variables, the prevalence of PCOS was consistently higher among women reporting the presence of symptoms compared to those without (**Figure 1**). The largest differences were observed for irregular menstrual cycles (78.2% *vs.* 23.8%) and hair growth in the jawline (83.5% *vs.* 37.9%). Notable differences were also seen for other hyperandrogenism-related features, including hair growth in the upper lip and thigh.

**Figure 2:**
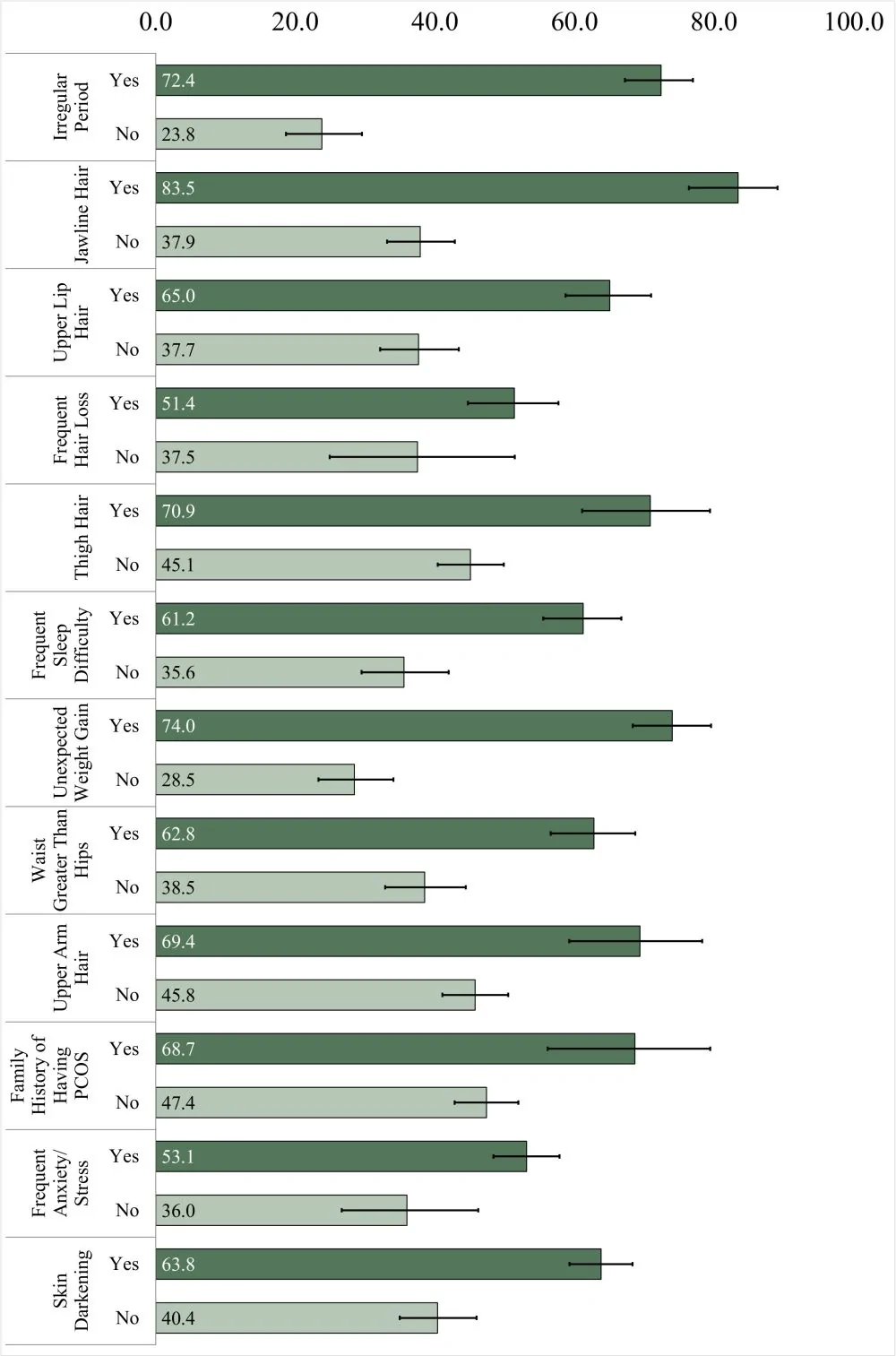
Prevalence of statistically significant symptoms from bivariate analysis among women with polycystic ovary syndrome (PCOS).

Among women with PCOS, the most frequently reported symptom was hair growth in the jawline (83.5%), followed by unexpected weight gain (74.0%) and irregular menstrual cycles (72.5%). High prevalence was also observed for hair growth in the thigh (70.9%) and upper arm (69.4%), along with a positive family history of PCOS (68.7%). Psychological symptoms were found to be comparatively less prevalent (**Figure 2**).

After adjusting for age and BMI, several variables remained significantly associated with PCOS (**Table 3**). Women with irregular menstrual cycles had nearly five times higher odds of having PCOS compared to those with regular cycles (OR = 4.93; 95% CI = 2.99–8.13, *P* < 0.001). Similarly, frequent unexpected weight gain was strongly associated with PCOS (OR = 4.32; 95% CI = 2.52–7.41, *P* < 0.001). Hair growth in the jawline also showed a significant association, with more than 4-fold higher odds of PCOS (OR = 4.25; 95% CI = 2.12–8.52, *P* < 0.001). In addition, women reporting frequent sleep difficulty had significantly higher odds of PCOS (OR = 2.62; 95% CI = 1.56–4.42, *P* < 0.001), and those with a waist circumference greater than hips had approximately 2.5 times higher odds (OR = 2.47; 95% CI = 1.51–4.02, *P* < 0.001). Although family history of PCOS showed a borderline association (OR = 2.10, *P* = 0.054), it did not reach statistical significance. Other variables, including darkening of skin, hair loss, acne, oily skin, anxiety or stress, fatigue, menstrual cramps, and hair growth in the upper lip, arm, and thigh, were not significantly associated with PCOS after adjustment.

**Table 3:** Multivariable logistic regression of symptoms associated with PCOS after adjusting for age and BMI

Variable	Category	OR	95% CI	*P*-value
Family history of having PCOS	No	Ref	-	-
	Yes	2.10	(0.99–4.49)	0.054
Irregular period	No	Ref	-	-
	Yes	4.93*	(2.99–8.13)	<0.001
Darkening skin	No	Ref	-	-
	Yes	0.83	(0.48–1.44)	0.507
Frequent hair loss	No	Ref	-	-
	Yes	1.33	(0.59–2.99)	0.493
Frequent unexpected weight gain	No	Ref	-	-
	Yes	4.32*	(2.52–7.41)	<0.001
Frequent acne breakouts	No	Ref	-	-
	Yes	0.92	(0.52–1.64)	0.783
Frequent oily skin	No	Ref	-	-
	Yes	0.71	(0.41–1.25)	0.236
Frequent anxiety/stress	No	Ref	-	-
	Yes	1.13	(0.58–2.19)	0.728
Frequent fatigue	No	Ref	-	-
	Yes	0.73	(0.36–1.47)	0.376
Frequent sleep difficulty	No	Ref	-	-
	Yes	2.62*	(1.56–4.42)	<0.001
Menstrual cramps	No	Ref	-	-
	Yes	1.43	(0.85–2.40)	0.178
Hair presence in upper lip	No	Ref	-	-
	Yes	1.36	(0.76–2.42)	0.311
Hair presence in jawline	No	Ref	-	-
	Yes	4.25*	(2.12–8.52)	<0.001
Hair presence in upper arm	No	Ref	-	-
	Yes	0.86	(0.37–2.01)	0.733
Hair presence in thigh	No	Ref	-	-
	Yes	1.48	(0.64–3.41)	0.362
Waist circumference greater than hips	No	Ref	-	-
	Yes	2.47*	(1.51–4.02)	<0.001

BMI – body mass index, CI – confidence interval, OR – odds ratio, PCOS – polycystic ovary syndrome, Ref – reference

*P < 0.001.

All variance inflation factors were below 4 (mean VIF = 1.84), indicating no evidence of substantial multicollinearity among the predictors. A sensitivity analysis using the original Likert scale categorical responses for the symptom variables yielded results consistent with the primary analysis in terms of the direction, magnitude, and statistical significance of associations.

## DISCUSSION

The objective of the current research was to compare the symptom profiles of women with and without PCOS, using non-clinical, self-reported and observable symptoms, in a Bangladeshi setting. The results show that there is a noticeable characteristic pattern of symptomatic features that distinguish women with PCOS and those without the disorder. The strongest association with PCOS was observed for irregular menstrual cycles. Moreover, metabolic characteristics, including unexpected weight gain and waist circumference greater than hips, were also strongly linked to the condition. Hyperandrogenism markers, particularly jawline hair growth, were also strongly associated. These findings indicate that a distinct combination of visible and self-reported symptoms is associated with PCOS status and may help identify women who could be at increased risk of the condition.

Irregular menstrual patterns were found to be the most significant distinguishing symptom of PCOS among the symptoms under analysis. The observation is in line with the pathophysiology of the condition, which is ovulatory dysfunction, which is a key element of PCOS, caused by hormonal imbalances that interfere with the normal development of follicles and ovulation [30,31]. As a result, menstrual irregularity is one of the main factors of the generally accepted Rotterdam diagnostic criteria [32]. The strength of association found in the present study is in line with the previous literature where irregular menstruation has been consistently cited as one of the most salient and dependable clinical signs of PCOS [33,34].

Hyperandrogenism markers, especially hair growth in the jawline and other androgen-sensitive areas, also showed significant independent relationships with PCOS. This observation is biologically plausible, since high androgen concentration is a characteristic of PCOS and it leads to the occurrence of hirsutism by promoting terminal hair growth in certain body parts [35,36]. The relatively high prevalence of jawline hair growth observed among women with PCOS in this study is consistent with prior literature suggesting that certain anatomical sites may be more sensitive to circulating androgens [37]. The literature has widely reported similar associations with hirsutism being regarded as a major clinical expression of PCOS [38]. Notably, these characteristics are observable externally without laboratory tests, which is especially useful in early diagnosis.

Metabolic features, especially unanticipated weight increase and waist circumference greater than hips were also discovered to be strongly linked with PCOS. These correlations are in line with the established role of insulin resistance in the pathophysiology of PCOS, which is a cause of metabolic dysfunction and elevated androgen production [39]. Central adiposity, which is indicated by a greater waist-to-hip ratio, is particularly significant because it is a sign of visceral fat buildup, which is a primary cause of metabolic risk [40,41]. The same pattern has been observed in previous research which indicated an increased prevalence of overweight status, central obesity and metabolic disturbances in women with PCOS [42]. These metabolic characteristics underscore the fact that PCOS is not just a reproductive abnormality but a wider metabolic health issue, such as the risk of developing type 2 diabetes and cardiovascular disease [43]. However, the association between unexpected weight gain and PCOS should be interpreted with caution. Given the cross-sectional nature of the study, it is not possible to determine whether weight gain preceded the development of PCOS or occurred as a consequence of PCOS-related metabolic disturbances.

Conversely, a number of symptoms that were frequently reported, such as acne, oily skin, hair loss, family history of having PCOS, fatigue, and anxiety, were not statistically significant after the possible confounders were taken into account. Though these symptoms have been previously reported to be associated with PCOS, these associations might not be consistent across populations because of genetic, environmental, and lifestyle differences [44–48]. These symptoms are also not very uncommon in the general population and may be affected by various non-specific factors which may decrease their discriminatory power in this regard. The non-significance of this study indicates that though these features might be included in the overall clinical presentation of PCOS, they are not as strongly associated as independent variables and must be viewed with caution especially in symptom-based screening methods.

### Implication of the findings

The findings of this study have important implications for the early identification of women who may be at increased risk of PCOS, particularly in resource-limited settings. The observed associations between several observable and self-reported symptoms and PCOS suggest that symptom-based approaches may have potential utility as an initial step in risk assessment. This may be particularly relevant in settings where access to hormonal testing, ultrasound examination, and specialist care is limited. However, further research is needed to develop and validate symptom-based risk assessment tools before their practical application can be recommended.

From a public health perspective, the findings suggest the potential value of community-based awareness and risk assessment initiatives based on simple, non-clinical indicators. Since several of the identified symptoms, including menstrual irregularity and visible signs of hyperandrogenism, can be recognised without specialised medical equipment, they may be incorporated into educational programmes, awareness campaigns, and primary healthcare settings. Such approaches could encourage timely healthcare-seeking behaviour and facilitate earlier clinical evaluation among women who may be at increased risk of PCOS.

Moreover, the findings provide a rationale for future research exploring digital health interventions that incorporate symptom-based risk assessment. Mobile health tools may offer a convenient platform through which women can assess their potential risk and seek appropriate medical advice. Such approaches may be particularly valuable in settings where social stigma or limited access to healthcare services create barriers to reproductive healthcare.

### Strength and limitations

There are a number of strengths in this study. The validity of the outcome measurement was increased by the use of physician-confirmed diagnoses to classify the cases, whereas the comparability and statistical efficiency were increased by the balanced sample size of the case and control groups. The study sample from various healthcare institutions helped to make the study population more diverse and representative. Moreover, the multidimensional evaluation of PCOS was possible due to the overall examination of a broad spectrum of symptoms, such as reproductive, metabolic, and androgen-related characteristics. The presence of trained physicians in the collection of data also enhanced the accuracy and reliability of the information collected. Data integrity was also ensured by the implementation of rigorous quality control procedures such as data validation and follow-up checks. In addition, a sensitivity analysis was conducted to assess the robustness of dichotomising the Likert-scale symptom variables, using the original categorical responses as predictors in the logistic regression model. The binary classification was retained in the final model for ease of interpretation and to preserve model parsimony.

There are a number of limitations that need to be taken into account when interpreting the results of this study. The use of self-reported symptoms can create bias in recall and inaccuracies in reporting. The assessment of hirsutism relied on participant self-classification using visual references rather than a validated clinical scoring system, which may introduce measurement error and inter-individual variability and could affect the strength of associations observed for hyperandrogenism-related symptoms. Moreover, the facility-based sampling strategy may limit the generalisability of the findings. Both cases and controls were recruited from tertiary healthcare institutions and may not fully represent women in the general population. Women seeking care at tertiary facilities may differ from community populations in terms of symptom severity, healthcare access, and underlying health characteristics. Consequently, the prevalence and relative importance of the identified symptoms may vary in other settings. Further community-based studies would help evaluate the generalisability of these findings to broader populations.

Although age and BMI were adjusted for in the multivariable analysis because of their well-established associations with both symptom presentation and PCOS, other factors may also influence the observed relationships. Variables such as physical activity, dietary habits, socioeconomic characteristics, family history of metabolic disorders, medication use, smoking, and reproductive health factors may affect both symptom occurrence and PCOS status. Inclusion of a large number of additional variables may reduce model parsimony and statistical efficiency; therefore, the final model focused on key confounding variables identified a priori. Nevertheless, residual confounding from unmeasured or uncontrolled factors cannot be completely excluded. Moreover, the cross-sectional study design does not allow evaluating the changes in symptoms and disease progression over time.

## CONCLUSIONS

This study identified a distinct pattern of observable and self-reported symptoms associated with PCOS, including irregular menstrual cycles, signs of hyperandrogenism, and metabolic characteristics. While not all commonly reported symptoms showed associations, the identified symptom profile highlights the potential value of symptom-based risk assessment approaches for identifying women at increased risk of PCOS. Further research is needed to develop and validate such approaches and to evaluate their feasibility, performance, and applicability in real-world settings.

Future studies might aim at confirming these results in larger and more heterogeneous populations, such as community-based samples. Longitudinal studies would be useful to determine the temporal association between the development of the symptoms and the diagnosis of PCOS. In addition, future studies could evaluate whether the identified symptom profile remains informative after accounting for a broader range of behavioural, socioeconomic, reproductive, and metabolic factors.

## Additional material


Online Supplementary Document

## Data Availability

**Data availability:** The data that support the findings of this study are available from the corresponding author upon reasonable request.
